# Laser melting manufacturing of large elements of lunar regolith simulant for paving on the Moon

**DOI:** 10.1038/s41598-023-42008-1

**Published:** 2023-10-12

**Authors:** Juan-Carlos Ginés-Palomares, Miranda Fateri, Eckehard Kalhöfer, Tim Schubert, Lena Meyer, Nico Kolsch, Monika Brandić Lipińska, Robert Davenport, Barbara Imhof, René Waclavicek, Matthias Sperl, Advenit Makaya, Jens Günster

**Affiliations:** 1 Faculty of Mechanical Engineering and Materials Science, Aalen University, Beethovenstraße. 1, 73430 Aalen, Germany; 2 Materials Research Institute Aalen, Aalen University, Beethovenstraße. 1, 73430 Aalen, Germany; 3 Federal Institute of Materials Research and Testing (BAM), Unter den Eichen 87, 12205 Berlin, Germany; 4 LIQUIFER Systems Group GmbH, Obere Donaustraße 97/1/62, 1020 Vienna, Austria; 5 Institut Für Materialphysik Im Weltraum, Deutsches Zentrum für Luft- und Raumfahrt (DLR), 51170 Cologne, Germany; 6 European Space Agency, ESTEC, Keplerlaan 1, P.O. Box 299, 2200 AG Noordwijk-ZH, The Netherlands; 7 Institute of Non-Metallic Materials, Clausthal University of Technology, Clausthal-Zellerfeld, Germany

**Keywords:** Aerospace engineering, Ceramics, Mechanical properties

## Abstract

The next steps for the expansion of the human presence in the solar system will be taken on the Moon. However, due to the low lunar gravity, the suspended dust generated when lunar rovers move across the lunar soil is a significant risk for lunar missions as it can affect the systems of the exploration vehicles. One solution to mitigate this problem is the construction of roads and landing pads on the Moon. In addition, to increase the sustainability of future lunar missions, in-situ resource utilization (ISRU) techniques must be developed. In this paper, the use of concentrated light for paving on the Moon by melting the lunar regolith is investigated. As a substitute of the concentrated sunlight, a high-power CO_2_ laser is used in the experiments. With this set-up, a maximum laser spot diameter of 100 mm can be achieved, which translates in high thicknesses of the consolidated layers. Furthermore, the lunar regolith simulant EAC-1A is used as a substitute of the actual lunar soil. At the end of the study, large samples (approximately 250 × 250 mm) with interlocking capabilities were fabricated by melting the lunar simulant with the laser directly on the powder bed. Large areas of lunar soil can be covered with these samples and serve as roads and landing pads, decreasing the propagation of lunar dust. These manufactured samples were analysed regarding their mineralogical composition, internal structure and mechanical properties.

## Introduction

To advance space exploration towards long-term missions, it will be crucial to use in-space manufacturing (ISM) and in-situ resource utilization (ISRU) technologies^1^. Given the extreme costs of shipping materials from Earth, a prerequisite for future human exploration will be the fabrication of objects directly on the surface of the Moon. Raw materials and energy can be harvested directly from the lunar surface, leaving equipment and some consumables the only necessity that must be brought from Earth^2^. The technology proposed in this project, the laser melting of a lunar regolith simulant (representing the energy of the sun and the lunar dust respectively) acts in place of existing resources for manufacturing on the Moon.

The generation of suspended dust when moving a vehicle across the lunar surface is a major risk for lunar exploration. When lunar dust is in contact with the exploration equipment, it can led to different damages on the instruments^3^. As an example, the study by Immer et al. investigated sandblast damage to the Lunar Surveyor III spacecraft. This damage was caused by lunar regolith ejection during the Apollo 12 landing^4^. Therefore, one of the first steps towards establishing a lunar base is the creation of infrastructure elements, such as roads for rovers and landing pads, as these constructions can help with dust mitigation. The main objective of the work presented in this paper is to produce elements with molten regolith that could be used during human and robotic lunar explorations to pave large areas and help to solve dust attenuation problems on the lunar surface. This technology is envisioned to play a major role in the first phase (survivability) of lunar infrastructure and base development^5^, and over time to contribute to all phases of lunar exploration: robotic lunar exploration, survival, sustainability, and operational phase^6, 7^.

Lunar regolith can be sintered or melted by intensive solar or laser radiation into dense, rigid structures^8^. To achieve the required energy densities, sunlight can be focused by means of lens and mirrors^9^. A major concern, however, is the quality of the manufactured parts. In addition, the construction of infrastructure requires a minimum productivity of the processes involved. Rapid densification of generally fluffy powders is associated with melting and viscous flow resulting in significant deformation of initially homogeneous powder beds. Light focused on small points is preferred to obtain parts with good geometric definition, but productivity is generally low.

With the aim of consolidating large structures from lunar regolith, the European Space Agency (ESA) project ‘PAVER’—Paving the road for large area sintering of regolith—studied whether the melting of regolith with a large beam of focused light is a suitable technology for paving applications on the Moon. The project assessed the time and power required to produce geometric shapes by laser melting with a large spot, the quality of the parts and the maximum size of the parts. To recreate the solar concentrated light in this study, a CO_2_ laser with a maximum power of 12 kW and spot diameter up to 100 mm was used. A lunar regolith simulant was used as raw material in this study. Different lunar regolith simulants are commercially available for ISRU studies^10, 11^. The candidate simulants for its use in this project were JSC-2A (developed by Zybec Advanced Production), LHS-1 (developed by Exolith), FJS-1 (by Shimizy Corporation) and EAC-1A (by ESA’s European Astronaut Centre). In this study, EAC-1A was selected as lunar regolith simulant due to its availability in large quantities.

### State of the art

Currently, different technologies for lunar manufacturing for in-situ lunar utilization are studied. Due to its fully automated nature, flexibility, and geometric freedom it offers, Additive manufacturing (AM) has been proposed for in-site fabrication on celestial bodies^12^ or under microgravity conditions^13^. One of these techniques, contour crafting (CC), would allow the construction of structures on the Moon using a lunar concrete. In the research carried out by Khoshnevis et al.^14^, the feasibility of CC combined with a robotic system for the automation of the process was studied. The team noted the difficulties of creating the adequate mixture on the Moon due to water constrains and mixing under lunar gravity conditions^14^. Following the CC research, Khoshnevis et al.^15^ investigated feasibility of sulphur concrete extrusion with different sulphur contents. In another study, Grugel et al.^16^ investigated the behaviour of sulphur concrete cubes subjected to lunar temperature cycles and reported that, after 80 cycles, material disintegration, due to the differences in thermal expansion coefficients of the constituting materials of the concrete, was macroscopically observed.

Another AM technology for lunar construction is binder jetting (BJ). In this technology, a binding agent is selectively deposited on to a bed of powdery material. Cesaretti et al.^17^ worked in their study with BJ for lunar applications. Furthermore, they applied vacuum (10^–3^ mbar) to the manufactured layers to simulate the lunar conditions. In this study, the samples reached 17–20 MPa of strength in the compression testing^17^. In addition, microwave sintering has been considered as another possibility for sintering of lunar regolith. Sintering consists of binding different particles of a powder by thermal diffusion of the material on an atomic scale, without melting, to obtain a solid structure. In this procedure, the heat produced by the electromagnetic wave is the thermal energy that facilitates the sintering of the powder. In the study of Lim et al., the microwave heating behaviour of JSC-1A was investigated under different microwave powers (250–1000W). They found that the fabrication of specimens at input powers 800–1000W generated homogeneous specimens in reasonable short time, however a cavity was used to concentrate the microwaves to the required energy densities^18^. Fateri et al.^19^ investigated and compared microwave sintered with oven sintered samples in their study.

In the evaluation and comparison of different lunar manufacturing methods carried out by Farries et al.^8^ the most promising technique discussed was regolith casting. This consists in heating up the lunar material inside a mold until the regolith is melted and then, cooling it down to obtain a fully dense sample. The composition of the lunar simulant and the cooling process followed, play a role on the degree of crystallization of the samples obtained^8^.

Looking at raw material and energy, solar energy and lunar regolith are directly available on the Moon, meaning that the direct sintering or melting of the regolith is a feasible approach for the manufacturing of objects on the lunar surface. Mobile solutions such as solar or laser energy sources can be used to manufacture roads or landing pads in-situ^8^. The first mention of the technology goes back to the year 1933, when Beach suggested to collect sunlight for the melting of sand for roads^20^. Cardiff and Hall tested the melting of lunar regolith simulant JSC-1A/JSC-1AF in laboratory environment in 2008^21^. They used a large quartz window to concentrate the solar energy and a large-aperture vacuum chamber to represent the lunar environment. An estimated sintering rate of 13 cm^2^/min indicated a production time of 55 days for a 100 m^2^ landing pad. The study by Woolf et al. describes the construction of a lunar habitat with a conical-shaped structure using a concentrated solar beam. The authors considered the use of a large paraboloid-shaped mirror as a sunlight concentrator, which should be of 50 m in diameter for the construction of a 2000 m^2^ habitat^22^.

In recent years, layer wise sintering of lunar regolith and relatively small-scale objects has been demonstrated using a laser^23–25^. Regolith undergoes densification by different mechanisms, from solid state to liquid phase sintering, to melting. Full or partial melting is especially attractive because it enables a rapid and selective densification of the regolith by means of focused light (laser or sunlight). However, it is commonly experienced that lack of control of the heat distribution as well as poor wetting (this is, poor contact of the liquid phase of molten regolith and the solid substrate) of the molten material yields a lack of fusion between the layers and ultimately a delaminated product^9^. Similar challenges for the solar sintering of regolith have also been described in other researches^26^. In general, the sintering and melting of powdery substrates to create dense structures by intensive laser radiation, is widely studied. In these processes, polymeric and metallic powders are fused to dense structures and a 3D object is built up layer by layer, such as in laser powder bed fusion (PBF-L)^27^.

Most related research employs focused laser beams with spot sizes of typically below 100 μm up to a maximum of some 100 μm^28^. The underlying concept is based on the fact, that structures with precise contours can be built up with small laser spots only. On the other hand, often these processes are clearly lacking productivity^29^. Moreover, the poor wetting of a fluffy powder bed by the melt results in the formation of individual drops rather than a closed surface. In this scenario, the interplay of surface tension of the melt and gravitational forces stipulate the shape of the molten material. Larger laser spots, however, form massive melt pools, which can be moved over the powder bed surface in a steady and continuous process, resulting in closed surfaces. Very little research has been devoted to the sintering of powders with laser spots as large as some 10–100 mm^9^. For the sintering of Lunar regolith in ISRU, however, such concepts are reasonable, as sophisticated optics capable of focusing light to small spots and realizing their fast movement over the sinterable powdery material are heavy and complex and thus difficult to carry into outer space.

## Results

### Determination of process parameters for laser melting

**Figure 1:**
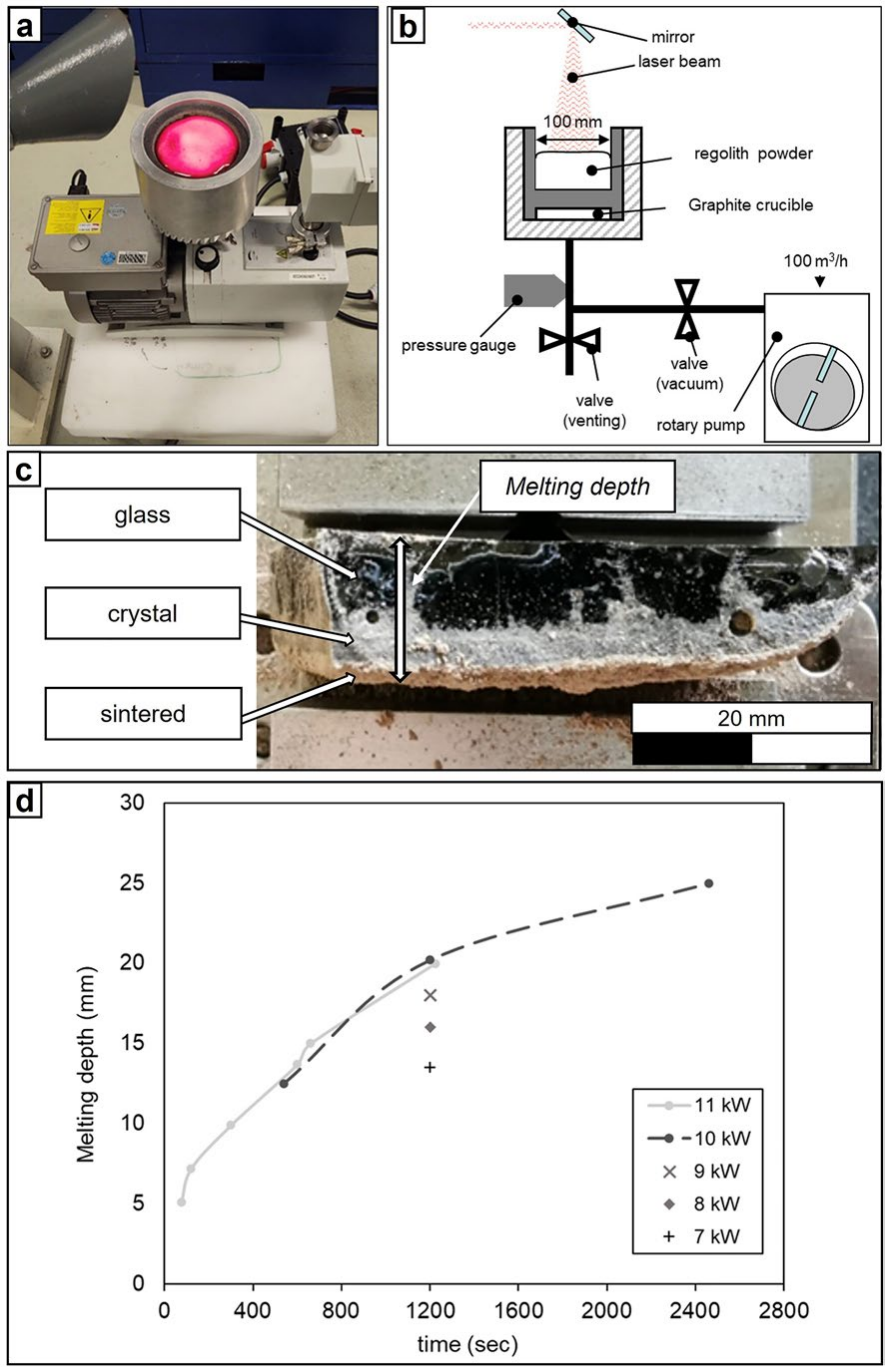
(**a**) Setup for the laser melting of EAC-1A with the possibility to apply vacuum. (**b**) Diagram of the application of vacuum thorough the powder bed. (**c**) Cross section of one fragment of a laser melted sample (10 kW, 600 s) showing the different material types within the sample. (**d**) Melting depth of EAC-1A powder bed as a function of laser radiation time. In addition, for a radiation time of 20 min (1200 s), different laser output powers have been applied.

A setup, consisting of a graphite crucible connected to a vacuum pump, was used to melt EAC-1A regolith simulant. This set-up is shown in Fig. 1a. The powder filled in the graphite crucible was irradiated by a laser with a spot diameter of 95 mm at different intensities and different dwell times to study the laser induced melting. The graphite crucible is equipped with porous graphite plugs at the bottom of the component, which makes it possible to apply vacuum (order of 10^–2^ mbar) from the bottom of the crucible throughout the powder bed. A diagram of the vacuum system can be seen in Fig. 1b. Therefore, this system allows the study of the melting depth as a function of time at reduced ambient pressure. In the experiment setup, the laser is incident from top, perpendicular to the powder surface. Figure 1c, shows the cross-section of one of the obtained samples. The top surface of the sample corresponds to the region directly affected to the laser, while the bottom of the sample was laying on the powder bed. From the cross-section of the sample, three different material regions can be noticed: a glass, a crystallized material and a thin layer of sintered material.

As a result of the fixed irradiating laser beam, the samples had a lens-like shape. The thickness of the obtained samples was measured at their center (maximum thickness). The melting depth was determined both as a function of the laser power output at a given time of irradiation and as a function of the time of irradiation at a given laser power output. The results obtained are summarized in Fig. 1d.

### Single and multi-track formation

**Figure 2:**
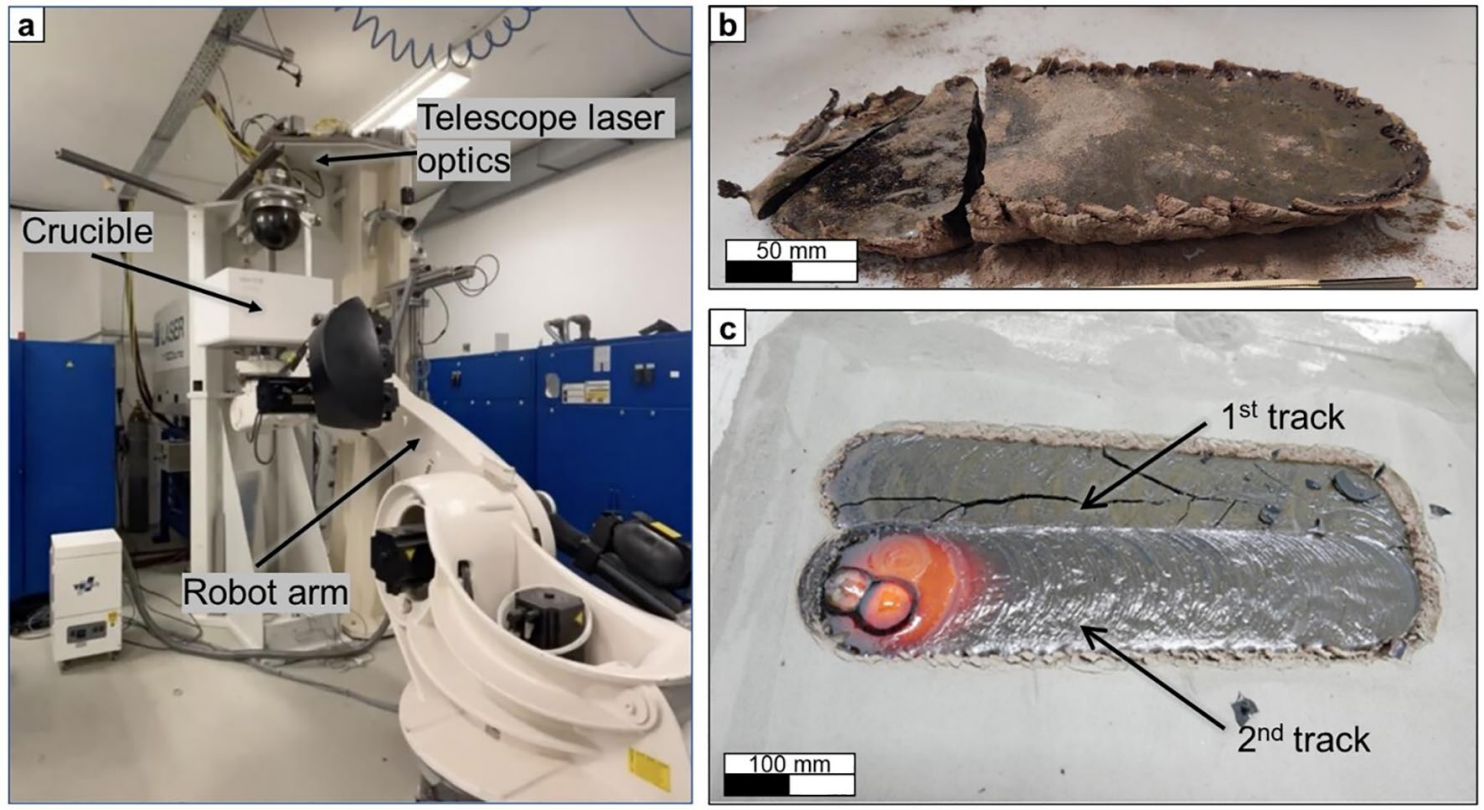
(**a**) Overview of the laser melting set up used. (**b**) One-track laser melted sample after manufacturing. (**c**) Two-tracks laser melted sample during manufacturing.

Initial experiments explored the laser melting of single and multi-track samples. The equipment consisted of a crucible filled with EAC-1A simulant which was attached to a robotic arm capable of moving the crucible under a fixed positioned CO_2_ laser beam. The crucible used was a commercial 14-inch quartz glass crucible for semiconductor use. No vacuum was applied in this case. Figure 2a, shows the setup used.

The EAC-1A powder was poured into the crucible and the laser irradiated a spot size of 95 mm in diameter with a laser power of 10 kW, while the crucible moved under the laser spot. The translation speed of the crucible and, thus, the speed of the powder bed was chosen to be 5 mm/min. This speed corresponded to an irradiation time of 1200 s in the center of the laser spot. Hence, according to the graph on Fig. 1, a melting depth of about 20 mm was expected and nearly reached at the center of the track. A maximum track length of 350 mm could be achieved in this experiment (Fig. 2b).

In the next step, two laser tracks were manufactured with 95 mm in width and a length of 500 mm and 18 mm thickness. The tracks had an overlap of 15% (Fig. 2c).

The manufactured sample analysis that is described later in the text, was performed using the laser-melted sample shown in Fig. 2c.

### Manufacturing of paving elements

**Figure 3:**
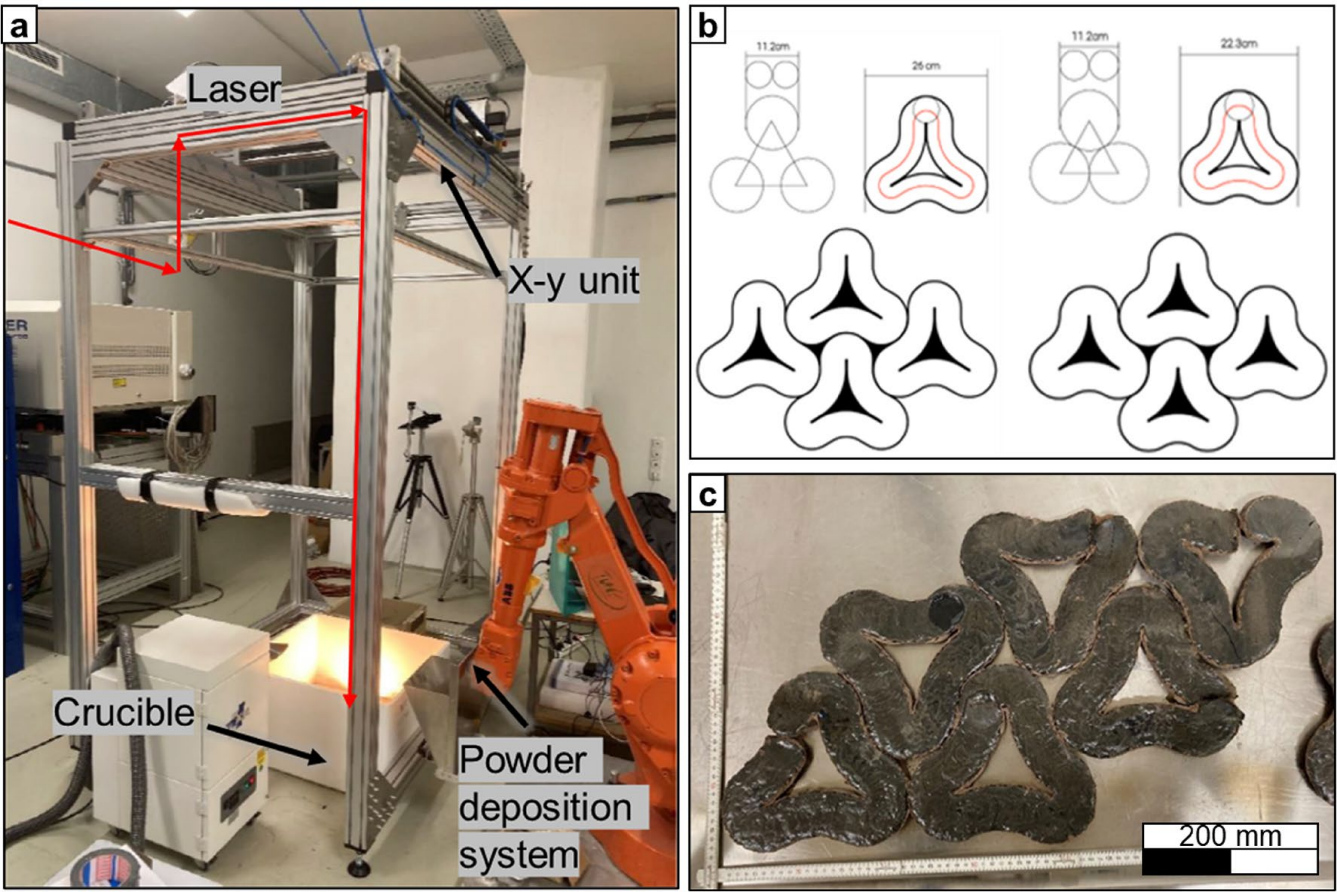
(**a**) Setup used for the manufacturing of interlocking geometries, (**b**) study of different interlocking geometries. (**c**) samples manufactured as paving elements.

In these experiments, the same CO_2_ laser was guided to an x–y axis setup, which made possible the movement of the laser spot over a stationary powder bed (Fig. 3a). This setup allowed a simple programming of complex shaped laser tracks, and the scannable area of the powder bed can be potentially enlarged up to 1 × 2 m. In this case, the geometries of the paving elements were designed following the principles of topological interlocking, which brings material’s flexibility, tolerance to local failures, and overall structural integrity without the need for any additional binders^30^.

Different scanning strategies were tested with the x–y-axis setup to create closed surfaces. An overlapping of laser tracks each time resulted in a cracking of the previously consolidated track due to internal thermal stresses. Hence, strategies avoiding overlapping were developed. Two final geometries were chosen and tested with a laser diameter of approximately 45 mm (Fig. 3b). A geometry trade-off associated with the geometries design process and the spot size of the laser was found, and a certain number of parts were produced. With the goal of making the interlocking capabilities of the samples higher, i.e., providing a smooth fit of the individual element, the design was iteratively adapted. An open structure of the samples with a hole in the middle was required in order to minimize overlapping of laser tracks and to provide a smooth interlocking. In this case, the spot diameter was 45 mm with a laser power of 3 kW and the laser incidence was perpendicular to the flat surface of the powder bed. The final samples manufactured in this case can be seen in Fig. 3c.

In a next step, the possibility of generating parts by multiple deposition of layers of molten regolith was explored. In these experiments, the laser was guided by the x–y axis set-up and the layers of regolith simulant were deposited with the powder deposition system shown in Fig. 3a. The deposited layers had a thickness of 15 mm. An additively manufactured sample can be seen in Supplementary Figure S1 online.

### Manufactured samples analysis

#### Computed micro-tomography

In Supplementary Figure S2, two images of the analysed sample are shown. In these images, it is possible to visualize the internal characteristics of the sample by its density contrast. In the bottom part of the sample, the layer of sintered material is observable as a darker thin area of material. The porosity was especially abundant in the crystallized material, while no porosity was visible in the region of glass. Furthermore, some cracks propagated in the glass area of the same sample are observed. These cracks may result from the rapid cooling of the glass during the fabrication process or be produced by the residual stress released during sample preparation.

#### Surface roughness

A fragment of the two-track sample was selected for the analysis of the roughness and surface profile parameters. From 5 measurements of surface roughness, the obtained arithmetical mean height (Ra) of the roughness profile was 43.6 µm with a standard deviation of 7.6 µm and the maximum height of the roughness profile (Rz) was 202.7 µm with a standard deviation of 24.9 µm.

#### Density

The density of the sample was calculated by dividing the mass and the volume of cubic probes obtained from the laser melted samples. The analysed samples were composed of both glass and crystalline material, as it was not possible to separate them mechanically without breaking the samples. Density measurements were performed for 4 samples (see values in Supplementary Table S1 online).

The average density of the samples was measured to be 2.76 g/cm^3^ with a standard deviation of 0.05 g/cm^3^. In the study carried out by Engelschion et al., the reported absolute density (the density of the individual grains) of EAC-1 powder was 2.9 g/cm^3^^31^. Considering this value, the porosity of the processed samples can be calculated to be approximately 4.8%.

#### Scanning electron microscope (SEM)

**Figure 4:**
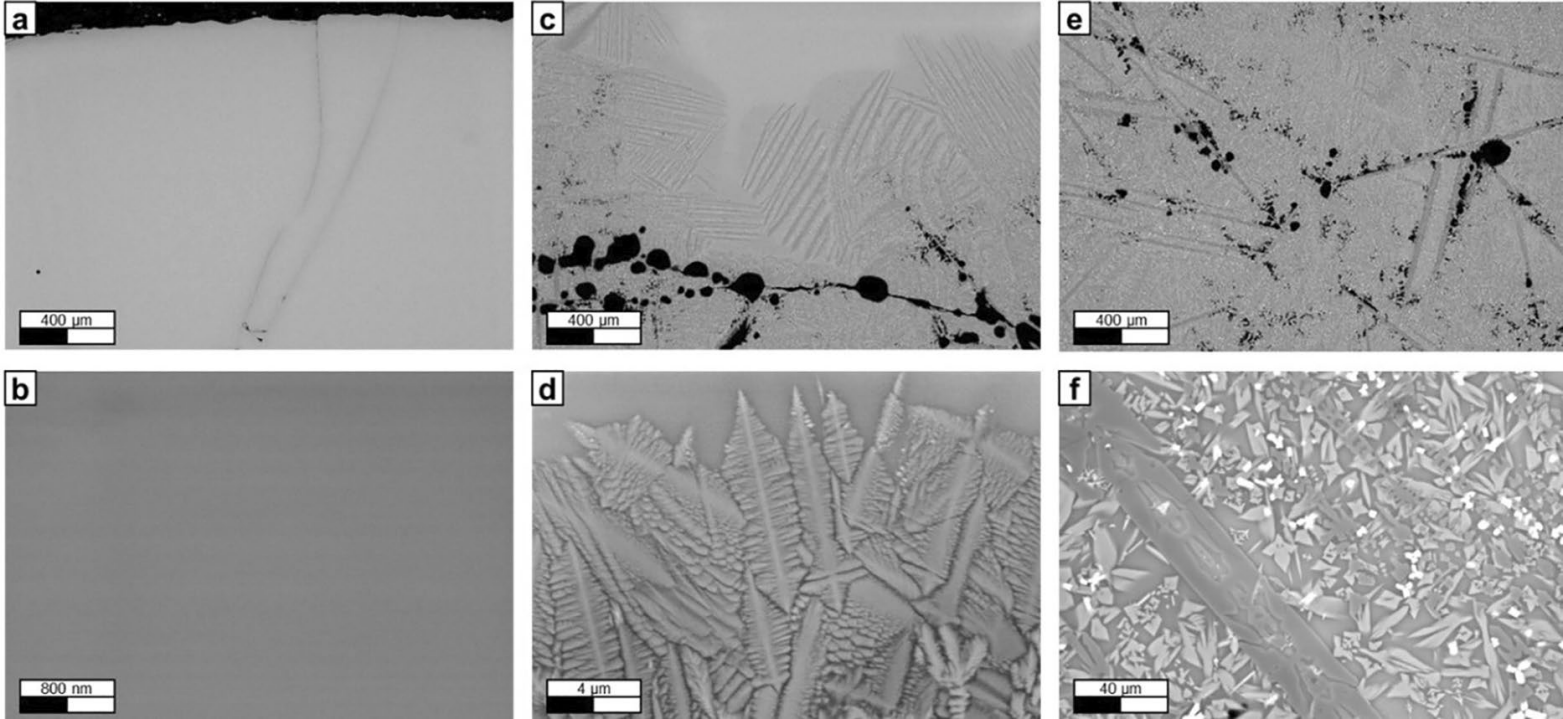
(**a**) SEM image of the glass phase (50X). (**b**) SEM image of the glass phase (30,000X). (**c**): SEM image of the crystalline-glass transition phase (50x). (**d**): SEM image of the crystalline-glass transition phase (5000X). (**e**) SEM image of the crystalline phase (50X). (**f**) SEM image of the crystalline phase (1000X). Images obtained with backscattered electron contrast.

The cross section of one of the fragments of the two-tracks laser melted sample was analysed with the SEM. The analysed areas were the glass and crystalline areas observed in the sample cross-section (Fig. 4).

In Fig. 4a, the glass phase can be observed. At this magnification (50X), no individual crystals are visible, and the material appears to be completely amorphous. In the centre of the image, the propagation of a crack is visible. In picture (b), a higher magnification (30,000X) of the glass area is shown with no indication of presence of microcrystals or nanocrystals. In (c), the area corresponding to the transition between glass material and crystalline material is analysed. Here, a group of voids of approx. 100 μm in average diameter are visible. In addition, a group of elongated crystals are observed in the glass-crystalline border. A high magnification image (5,000X) of the transition phase can be seen in (d). In this image, the dendritic shape of the microcrystals is detected.

Figure 4e shows a group of disperse thin crystalline structures (some of which are longer than 200 μm). These structures are surrounded by small crystals which are difficult to distinguish at this magnification. Furthermore, some voids of approx. 50 μm in diameter are visible (many of which are formed in the edges of the elongated crystalline structures). The detailed view of small crystals is shown in (f), in this image the magnification is 1000X. In this picture, a layer of elongated crystalline structure is visible, as well as groups of smaller crystals of different compositions. The different colours of the crystals are due to their different chemical composition, and therefore, different mineral phases in the sample.

#### Energy disperse X-ray (EDX)

For EDX analysis, three measurements were performed on the same fragment analysed with the SEM: the analysis of the composition of different individual phases (spots) were carried out for the glass area, crystalline area and sintered area (see Fig. 1c). In the EDX analysis of the glass phase, a high abundancy of oxygen (36.6 wt%) and silicon (21.2 wt%) in the material can be observed. Iron is the third element with highest concentration in the glass phase (10.2 wt%). Magnesium and calcium are present in similar quantities (8.6 and 8.8 wt%, respectively). Other metals (aluminium, sodium, titanium, and potassium) are present in lower amounts.

In the analysis of the crystalline phase, a measurement was performed by selecting individual spots on the sample’s surface. In this analysis, three groups of crystals were identified: forsterite, augite and an iron-rich phase that could not be associated with a specific mineral. Regarding the composition of the sintered phase three groups of crystals were identified: anorthite, augite and an iron-rich phase that could not be associated with a specific mineral. Two images of the analysed spots can be seen in Supplementary Figure S3 and S4 online.

#### X-Ray diffraction analysis (XRD)

**Figure 5:**
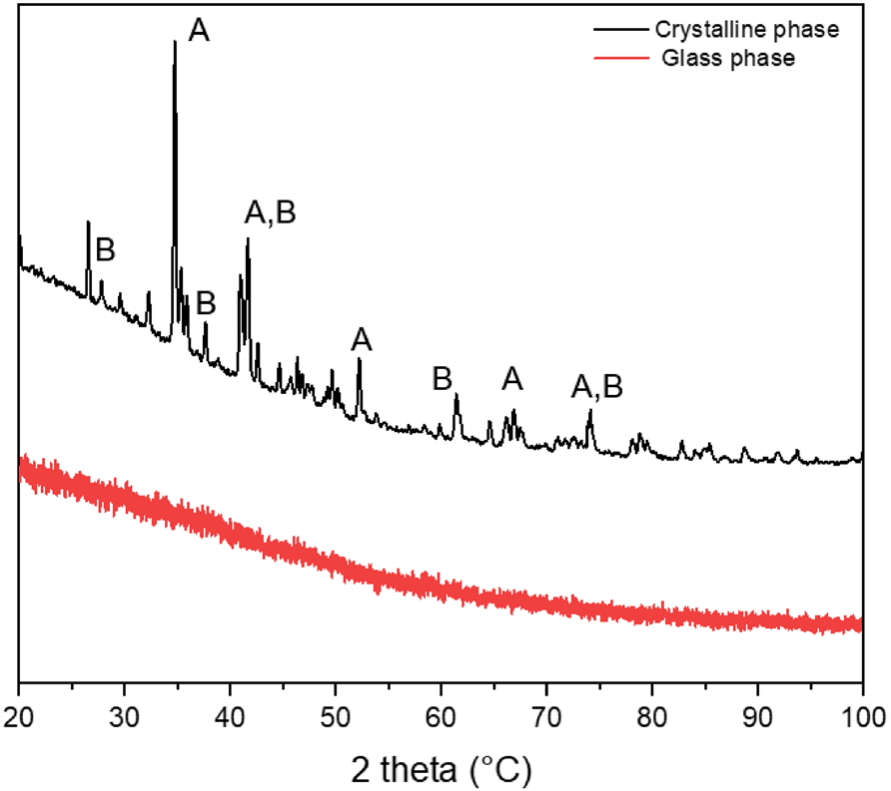
Diffraction pattern of the crystallized area (top). Diffraction pattern of the glass powder (bottom). Major peaks are labelled: (**A)**, augite; (**B**), silica.

The laser melted sample was further analysed using XRD. The aim was to identify the crystallographic structure of the existing phases found by EDX. Two areas of the material were analysed: first, the crystallized area and second, the glass area of the sample. For this purpose, a fragment of the glass material was cut from the laser melted sample. For the analysis of the glassy material, the glass fragment was grinded to obtain a powder out of it, which was subsequently examined with the XRD device. In Fig. 5, the diffraction pattern of the analysed crystalline area and the glass powder is shown.

The results show that the phases found are augite (magnesium iron calcium aluminium silicate) and silica (SiO_2_). In the diffraction pattern of the glass powder, no peaks are visible, as expected for an amorphous material.

#### Compression strength

For compression tests, ten cubic samples of 10 × 10 × 10 mm were used. These samples included both the crystalline and glass phase as it was not possible to separate them mechanically without breaking the sample. The compression experiments were performed using a Zwick Z100 machine and the sample’s compression strength was measured. The compressive strength of the samples varied between 216.29 and 56.19 MPa with a mean value of 93.97 MPa, with standard deviation of 55.88 MPa.

## Discussion

The results of the ESA project called PAVER are presented in this paper. In addition to this work, a more detailed study on the characterization of this simulant for its use in laser melting or sintering fabrication was carried out in the frame of the PAVER project. In this study, a water content of 1.2% was found in the EAC-1A powder. Prior to the laser melting experiments, the powder was dried at 180 °C (during 3 days for 10 kg of material) to remove the absorbed water from the powder and prevent the formation of bubbles in the melt due to water evaporation.

In the first step of the project, the relation between laser power output, irradiation time and melting depth was investigated. Regarding this, the EAC-1A contained in a crucible was irradiated with a CO_2_ laser at different laser power and dwell times and the resulting melting depths were measured. According to the laser power output and irradiation time graph, a maximum melting depth of 25 mm was reached after 2400 s. As expected, the melting depth saturated as the time increased. At 1200 s a melting depth of 20 mm was observed, doubling the time to 2400 s just resulted in a 25% increase in melting depth. In addition, laser powers of 11 kW and 10 kW at the same spot size of 95 mm resulted in the same depth, indicating that excessive laser power did not result in an increase in surface temperature of the melt. The surface temperature at this laser intensity (laser power per unit area), that was 142 W/cm^2^ for 10 kW, was 1600 °C. The temperature was not significantly increasing for 11 kW, respectively 157 W/cm^2^, which was 1640 °C. Lower laser powers resulted in a lower melting depth. At 7 kW, respectively 100 W/cm^2^, a surface temperature of 1350 °C was reached. Therefore, a laser power of 10 kW was selected for the manufacturing of large 2D geometries.

The next step of the project was the fabrication of 2D samples of lunar regolith simulant. The initial geometries were developed to test relatively simple shapes, able to be created with a single laser path. The first geometry tested consisted of two laser tracks melted next to each other with an overlap of 15%. The material was poured into a larger crucible and the laser irradiated a spot size of 95 mm with a laser power of 10 kW, while the crucible was moving under the laser spot at 5 mm/min. This speed corresponds to an irradiation time of 1200 s in the centre of the laser spot. Hence, a melting depth of approximately 20 mm was expected. However, the initial molten and solidified track was cracked while the second track was being formed. The reason behind such a crack formation could be explained by the induced thermal shock to the already solidified first track, during melting of the second track. It was also noticed that an increase in the laser speed led to the formation of cracks in the samples and to its final breakage. This phenomenon can be explained due to the relatively fast cooling rate of the samples and the resulting thermal shock induced in the material. It is noteworthy, that not the dwell time at maximum temperature in the laser spot is the critical parameter to avoid crack formation, but the cooling rate at the tailing edge of the laser spot.

As such, a different geometry with no overlaps was tested to reduce the risk of crack formation due to thermal shock. In this experiment, a geometry with interlocking capabilities was produced. For this, the x–y set up was used since it facilitated the programming of complex interlocking laser paths. Furthermore, due to the design of the interlocking samples, the laser spot diameter was reduced to approximately 45 mm. For this reason, the laser power was consequently reduced to 3 kW (intensity of 188 W/cm^2^), to maintain a laser intensity similar to the previous experiment but increased approximately 20% to compensate heat loses at the edge of the laser spot. The laser speed was kept at 5 mm/min, same as for the 95 mm laser spot, corresponding to 540 s exposure in the centre of the track. The reduction of the exposure time resulted in a lower melting depth. With this geometry, a certain number of elements has been produced, in order to test their interlocking capabilities. However, in some cases, the subsequent development of cracks caused by internal residual stresses was observed. These samples had a length of approximately 220 mm per side.

Finally, an additively manufactured sample was fabricated using the powder deposition system for the spreading of layers of EAC-1A simulant on top of the previously consolidated layer of material. The previously described geometry with interlocking capabilities and the same laser parameters as in one-layer manufacturing were used in this case (3 kW, 5 mm/min). The laser was guided with the x–y set up. A three-layer sample was obtained, with a total thickness of 22 mm. However, cracks developed in the previously consolidated layer of material during the melting process of the next layer of material. Therefore, it was not possible to create a non-defect additively manufactured sample with these experiment conditions.

The cross section of the laser molten samples obtained in the process showed a dense but glassy phase. As expected, the melting depth was not uniform, but higher in the middle of the track. At the top surface region of every sample, the material was fully glassy, in lower regions a dendritic crystalline phase was observed and at the frontier to the not yet molten powder a thin layer of sintered material can be recognized. The glass material in the top of the samples was a consequence of the high cooling rate of the molten material by heat radiation at the sample’s surface, while a lower cooling rate of the molten material surrounded by non-molten powder, facilitated the crystallization on this material region.

The roughness of the glassy top surface of the samples was measured, and while the relatively low value obtained (Ra = 43.6 µm) could negatively influence the driving performance of an exploration vehicle, for a better understanding of the driving performance, complementary research on the surface-wheel friction needs to be performed and the surface sample should be further characterized (since, for example, the waviness of the profile could have an influence in the friction^32^).

The presence of microcrystals or nanocrystals embed in the glass phase was investigated using SEM at different magnification. No such elements were found. Furthermore, the mineralogical compositions of the crystalline and glass phases were studied using EDX. A uniform composition was detected for the glass phase and different minerals were observed in the crystalline and sintered regions. In order to corroborate these results, an XRD analysis was performed both for the crystalline phase and to the glass. The XRD results matched the information obtained by EDX, in which augite was a detected mineral in several of the analysed crystalline sample areas. As illustrated in Fig. 5, no peaks are visible in the diffraction pattern of the glass phase. This absence of diffraction peaks is assigned to an amorphous material in which the atoms of the material are randomly oriented. The presence of a glass layer on the surface of the paving elements could limit their field of application, as the glass could be damaged when subjected to strong thermal shocks (e.g., during rocket lift-off if the paving elements cover the launch pad). If that is the case, sharp fragments of broken glass could be generated posing a risk to the space mission. Further research should be carried out to study the thermal shock resistance of the samples, as well as the crack behaviour when subject to thermal changes.

Since in practice regolith processed using this technique will be required to support the weight of heavy exploration equipment, the compressive strength of the samples was analysed and then compared with the results of the RegoLight project in which the samples were manufactured using concentrated solar energy for sintering^33^. The average compressive strength of the laser melted samples (93.97 MPa) was approximately 50 times higher than the ones obtained in the RegoLight project (2.49 MPa). The values obtained in this project were similar to the compressive strength of concrete, which are in the range of 20–120 MPa^34^. However, a considerable standard deviation was noticed in the results of the compressive strength of the samples. These non-homogeneous results could be related to the internal defects (porosity and micro cracks) that were detected inside the samples by Computed Micro-tomography and SEM.

## Conclusions

In this paper, a technology scenario comparable to the use of concentrated sunlight for paving on the Moon was presented. A high-power CO_2_ laser was used to simulate a sun-light concentrator and a lunar regolith analogue was used to replicate the lunar soil. Furthermore, the large beam spot used (up to 100 mm) allowed the fabrication of large samples with a high thickness. The results of this study proved the viability of this technique for manufacturing large samples with interlocking capabilities in-situ that can be fabricated directly on the lunar surface and arranged for paving applications (without the need of additional manufacturing equipment like furnaces or moulds). For the application scenario of this technology on the Moon, a sunlight concentrator could be transported from Earth and deployed on the Moon. Using a Fresnel lens as a light concentrator and considering the required power (188 W/cm^2^) and the spot area (0.16 m^2^ with a spot diameter of 45 mm) for the fabrication of the interlocking elements, this would translate into a lens of approximately 2.37 m^2^ (considering a solar flux on the Moon of approximately 1400 W/m^2^^35^ and an efficiency of the lens of 90%). The relatively small size of the required equipment and the simplicity of the system would be an advantage for the use of this technology in future missions on the Moon.

## Methods

### Material

The lunar regolith simulant EAC-1A is used in this study. In a study carried out by Engelschiøn et al.^31^ the mineral content of EAC-1A was a 55% plagioclase, 25% olivine and 10% pyroxene. The chemical composition of EAC-1A consists of SiO_2_ (43.7%), Al_2_O_3_ (12.6%), Fe_2_O_3_ (12%), MgO (11.9%) and CaO (10.8%) according to the same research.

### Computed micro-tomography

A ‘MicroCT 42’ system from Scanco Medical AG (Bassersdorf, Switzerland) was used at 70 kV, 114 μA with a voxel size of 18 μm. For the determination of volume of the parts, VG Studio Max 3.2, (Heidelberg, Germany), a commercial software for XCT visualisation and measurement was used.

### Surface roughness

The measurements were done by following ISO 1997/2009 norm using a Surfcom Touch 50 instrument from Accretech Tokyo Seimitsu Co., Ltd. (Japan). Five different measurements were performed on the fragment. The selected measurement parameters were: λc = 8 mm, λs = 2.5 μm, v = 1.5 mm/s, L = 40 mm.

Where λc is the cut-off wavelength of the high-pass filter, λs f is the cut-off wavelength of the low-pass filter, v is the measurement speed and L is the evaluation length.

### Density

The volume is calculated as $V = a \times b \times c$, where *a, b, c* are the sizes of the edges of the cube. The size of edges was measured with a digital calliper (± 0.01 mm). The density is given as $d=\mathrm{m}/\mathrm{V}$; where m is the mass of the sample. The mass was measured on a laboratory scale (± 0.01 g).

### Scanning electron microscope (SEM)

The sample was grinded and polished using a Struers Tegramin-30 semi-automatic grinding/polishing machine. Afterwards, the sample was studied using a Zeiss Sigma 300 VP microscope. Parameters of the analysis:

- Figure 4a,c,e: EHT = 20 kV and WD = 8 mm.
- Figure 4b,d: EHT = 10 kV and WD = 5.7 mm.
- Figure 4f: EHT = 20 kV and WD = 7.9 mm.
- Backscattered electron detector.

### Energy disperse X-ray (EDX)

A Zeiss Sigma 300 VP microscope equipped with an AMETEK EDAX 30 mm^2^ EDS detector was used for this analysis. Experiment conditions: EHT = 20 kV.

### X-ray diffraction analysis (XRD)

The diffraction patterns were collected with the GE “Seifert Analytic XRD Sun” diffractometer, XRD Eigenmann GmbH, formerly distributed by GE (Co radiation 50 kV, 35 mA). The angular range in 2-theta was 20–100° with a step size of 0.013° and a measuring time of 400 or 200 s. The software used for the identification of the phases was Match!. Database: COD (Crystallography Open Database).

### Compression strength

For compression tests, ten cubic samples of 10 × 10 × 10 mm were cut with an EXAKT 300/30 device using a diamond band. The speed of the diamond band was set to 100 m/min. The compression experiments were performed using a Zwick Z100 machine. ISO17162/2014 was followed in these experiments.

### Supplementary Information

Supplementary Information.

## Data Availability

The datasets used and/or analyzed during the current study are available from the corresponding author on reasonable request.
