# Supplementary material for: Laser melting manufacturing of large elements of lunar regolith simulant for paving on the Moon
Source: Sci Rep. 2023 Oct 12;13:15593. doi: 10.1038/s41598-023-42008-1 (PMC10570301; doi:10.1038/s41598-023-42008-1)
Supplement: Supplementary file 1 — Supplementary Information. [file 41598_2023_42008_MOESM1_ESM.docx]

**Laser melting manufacturing of large elements of lunar regolith simulant for paving on the Moon**

**Juan-Carlos Ginés-Palomares^1^*, Miranda Fateri^1^*, Eckehard Kalhöfer^1^, Tim Schubert^2^, Lena Meyer^3^, Nico Kolsch^3^, Monika Brandić Lipińska^4^, Robert Davenport^4^, Barbara Imhof^4^, René Waclavicek^4^,** **Matthias Sperl^5^, Advenit Makaya^6^, Jens Günster^3,7^***

^1^Faculty of Mechanical Engineering and Materials Science, Aalen University, Beethovenstraße. 1, 73430 Aalen, Germany.

^2^Materials Research Institute Aalen, Aalen University, Beethovenstraße. 1, 73430 Aalen, Germany.

^3^Federal Institute of Materials Research and Testing (BAM), Unter den Eichen 87, 12205 Berlin, Germany

^4^LIQUIFER Systems Group GmbH, Obere Donaustraße 97/1/62, 1020 Vienna, Austria

^5^Institut für Materialphysik im Weltraum, Deutsches Zentrum für Luft- und Raumfahrt (DLR), 51170 Cologne, Germany

^6^European Space Agency, ESTEC, Keplerlaan 1 - PO Box 299, 2200 AG Noordwijk-ZH, The Netherlands

^7^Clausthal University of Technology, Institute of Non-Metallic Materials, Clausthal-Zellerfeld, Germany

**Supplementary information**

**Manufacturing of paving elements**


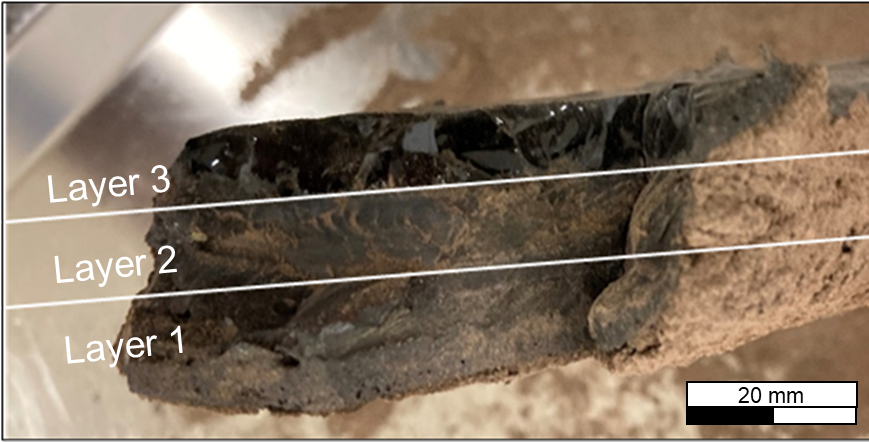


Supplementary Figure S1. Cross section of a part made by depositing two additional layers (15 mm thickness) on top of the initial structure in the powder bed.

**Computed Micro-Tomography**


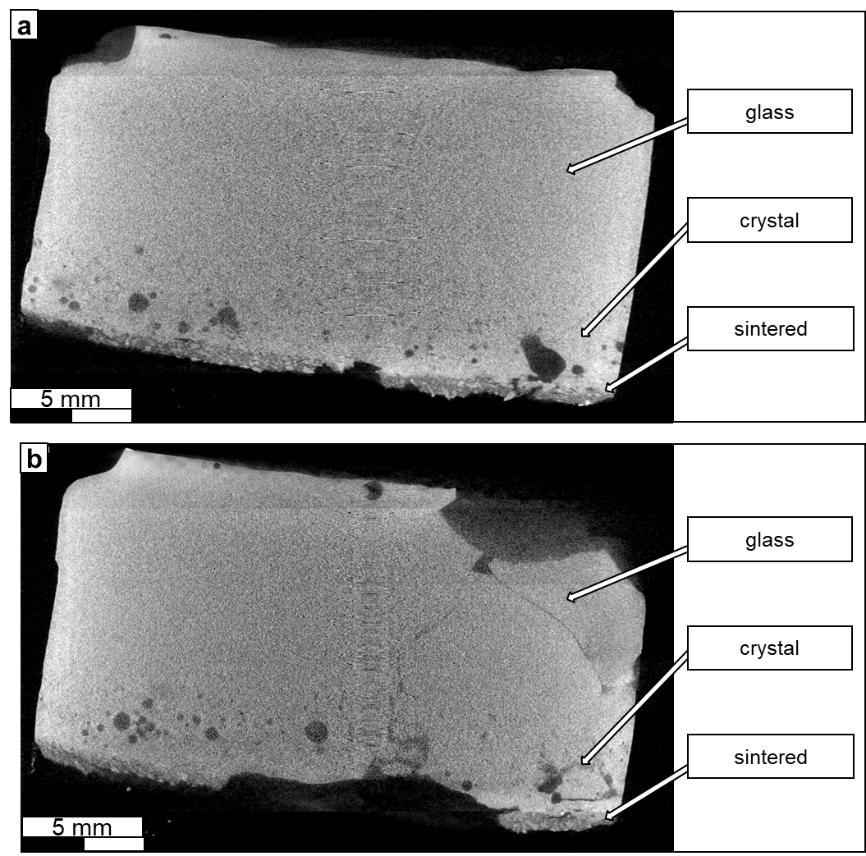


Supplementary Figure S2. (a,b) Images of the Micro-tomography of a laser melted sample.

**Density**

In Supplementary Table S1, the mass of the samples, the edge parameters (*a, b, c*), volume and value of the obtained density are listed. The average density of the samples is 2,76 g/cm^3^ (0,054 g/cm^3^ standard deviation).

Supplementary Table S1. Parameters and density values of the samples.

|  | **Sample 1** | **Sample 2** | **Sample 3** | **Sample 4** |
| --- | --- | --- | --- | --- |
| *m* (g) | 2,51 | 3,047 | 2,89 | 2,42 |
| *a* (mm) | 9,74 | 9,71 | 10,69 | 10,04 |
| *b* (mm) | 10,24 | 11,23 | 10 | 9,58 |
| *c* (mm) | 9,32 | 10,21 | 9,69 | 8,9 |
| *V* (mm^3^) | 929,5 | 1113,3 | 1035,9 | 856,03 |
| *d* (g/cm^3^) | **2,70** | **2,74** | **2,79** | **2,82** |

**Energy Disperse X-ray (EDX)**


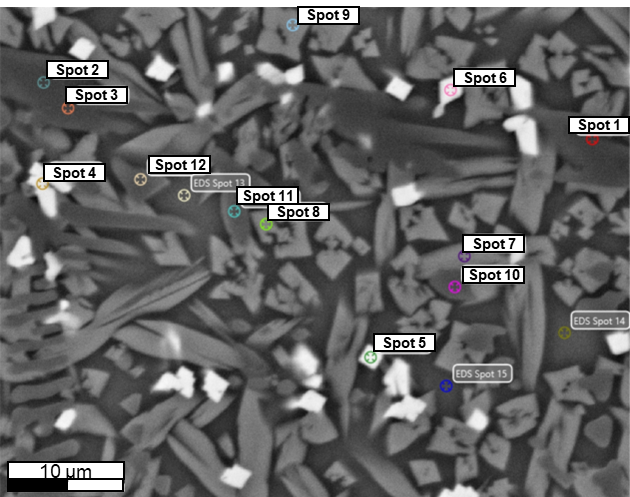


Supplementary Figure S3. Image of the spots analysed in the crystalline region.

Crystalline region analysis. Spots in Supplementary Figure S3. Image of the spots analysed in the crystalline region.

Spot1, spot2, Spot3, Spot10, spot11 and spot12: Forsterite.

Spot4, spot5 and spot6: Iron rich.

Spot7, spot8 and spot9: Augite.

The minerals of other spots analyzed in this region could not be identified.


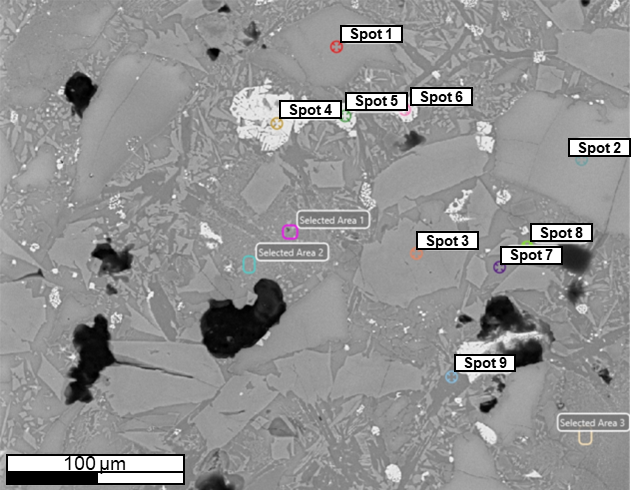


Supplementary Figure S4. Image of the spots analysed in the sintered region.

Sintered region analysis. Spots in Supplementary Figure S4

Area1, area2 and area3: Augite.

Spot4, spot5 and spot6: Iron rich

Spot7, spot8 and spot9: Anorthite.

The “Selected areas” shown in Supplementary Figure S4 were not considered as regions of interest in this analysis.
